# Treatment outcomes of squamous cell carcinoma of the external auditory canal and potential benefit of induction chemotherapy followed by chemoradiotherapy

**DOI:** 10.3389/fonc.2025.1530922

**Published:** 2025-08-15

**Authors:** Yosuke Mizunari, Masato Nagaoka, Naohiro Takeshita, Kazuki Kanno, Haruyuki Hirayama, Taisuke Akutsu, Hisashi Kessoku, Katsuhiro Ishida, Yutaka Yamamoto

**Affiliations:** ^1^ Department of Otolaryngology, The Jikei University Kashiwa Hospital, Kashiwa, Japan; ^2^ Department of Otolaryngology, The Jikei University School of Medicine, Tokyo, Japan; ^3^ Department of Plastic and Reconstructive Surgery, The Jikei University School of Medicine, Tokyo, Japan

**Keywords:** squamous cell carcinoma, external auditory canal, revised Pittsburgh classification, overall survival, disease-free survival, induction chemotherapy

## Abstract

**Introduction:**

Carcinoma of the external auditory canal (EAC) is rare, and squamous cell carcinoma (SCC) is the most common histological type. There are few reports on the treatment outcomes for a large number of cases at a single institution, and a standard treatment has not been established.

**Methods:**

Treatment details and prognoses were retrospectively examined for patients who underwent primary treatment for SCC of the EAC at The Jikei University between April 2015 and May 2023.

**Results:**

Twenty-seven patients with SCC of the EAC were included (median age of 64 years). Analysis using the revised Pittsburgh classification revealed that there were 3 cases of T1, 4 cases of T2, 9 cases of T3, and 11 cases of T4. Among the patients, 13 were treated surgically, 1 underwent partial resection of the EAC, 11 underwent lateral temporal bone resection, and 1 underwent subtotal temporal bone resection. The remaining 14 patients received nonsurgical treatment: 1 with radiotherapy, 3 with concurrent chemoradiotherapy, and 10 with induction chemotherapy (ICT). The overall survival (OS) and disease-free survival (DFS) rates at 3 years were 72.8% and 50.5%, respectively. When the surgical and non-surgical groups were compared, the 3-year OS and DFS rates were 92.3% and 68.3% for those who underwent surgery and 47.6% and 35.7% for those not treated with surgery, respectively, suggesting a better prognoses for patients who underwent surgical treatments (*p* = 0.045, 0.052). In the non-surgical group, the 3-year OS and DFS rates were 90.0% and 50.0% for those who received ICT and 0% and 0% for those who did not receive ICT, respectively, indicating better prognoses for patients treated with ICT (*p* = 0.0075, 0.0012).

**Conclusion:**

At our institution, the 3-year OS and DFS rates of patients with SCC of the EAC were favourable for those who underwent surgery and received ICT. These findings suggest that treatment outcomes can be improved by using ICT in nonsurgical treatments for patients with SCC of the EAC.

## 1 Introduction

Carcinoma of the external auditory canal (EAC) is rare, with an incidence of approximately 1 case in 1 million people (1). The most common histological type of EAC cancer is squamous cell carcinoma (SCC), which accounts for 80% of all cases (2). Other types, such as basal cell carcinoma and adenoid cystic carcinoma, have also been reported (3). Because of the rarity of this cancer, few studies have documented treatment outcomes among a large number of cases at a single institution, and a standard treatment protocol has not been established. Currently, the extent of tumour spread is evaluated using the internationally accepted revised Pittsburgh classification system (4). Several previous studies have investigated treatment outcomes, but there is no consensus on standard therapy (5, 6).

Treatment strategies include surgery and chemoradiotherapy (CCRT). Standard surgical procedures for carcinoma of the EAC include lateral temporal bone resection (LTBR) and subtotal temporal bone resection (STBR), or a combination of these procedures (4). The EAC, a part of the temporal bone, is in close proximity to vital structures such as the inner ear, facial nerve, sigmoid sinus, carotid artery, and middle and posterior cranial fossa, thereby complicating surgery, even for early-stage carcinomas, and limits the number of centres capable of offering treatment (7). Notably, STBR is a highly invasive procedure, which further increases surgical complexity (8). In a systematic review of 437 patients with SCC of the EAC, 50 underwent surgery, 160 underwent surgery followed by adjuvant radiation therapy, 190 were treated with radiation therapy alone, and only 35 received chemotherapy (9). Additional studies reported the efficacy of concurrent CCRT and preoperative chemotherapy (6, 7, 10); however, studies focusing on induction chemotherapy (ICT) in conjunction with CCRT are limited (11). We advocate surgery as the initial treatment for operable cases of SCC of the EAC and reserve radiotherapy (RT) for cases when surgery is unfeasible or undesired.

In this study, the demographics, treatment approaches, and post-treatment outcomes of patients with SCC of the EAC treated at our department were reviewed. Overall survival (OS) and disease-free survival (DFS) rates were retrospectively analysed based on disease outcomes.

## 2 Materials and methods

This study included patients with EAC carcinoma who visited The Jikei University School of Medicine and Kashiwa Hospital between April 2015 and May 2023 and were placed on a curative treatment plan. Eligible patients were those who were pathologically diagnosed with SCC and completed treatment at our hospital.

From the patients’ medical records, we extracted basic patient information (age and sex), chief complaints, pre-existing medical conditions, disease staging, treatment details (surgical techniques, chemotherapy, and CCRT details), resection margins, post-treatment status, and disease outcomes until April 2024. Tumour extension at the primary site was evaluated using the revised Pittsburgh classification, whereas cervical lymph nodes and distant metastases were assessed according to Union for International Cancer Control criteria. Treatment-related toxicity was evaluated using the Common Toxicity Criteria for Adverse Events (CTCAE) (version 4.0). Survival estimates were calculated using Kaplan–Meier method, and the log-rank test was used to compare survival rates. Hazard ratios (HRs) were determined using the Cox proportional hazards model. Statistical analyses were performed using STATA/Basic Edition (version 18.0; StataCorp., LLC, College Station, TX, USA), with the significance level set at *p* < 0.05.

This study was approved by the Ethics Committee of Jikei University School of Medicine (approval No. 35-059 (11682)).

### 2.1 Treatment strategy

According to the revised Pittsburgh classification, LTBR is the preferred approach for treating T1 and T2 tumours. For T3 and T4 cases, LTBR or STBR was the treatment of choice for patients without dural or extensive mastoid sinus involvement, allowing for complete resection through a combined approach involving the inner ear, facial nerve, and both middle and posterior cranial fossae. Surgical procedures on the middle cranial floor were performed in collaboration with neurosurgeons. Plastic surgeons determined the reconstruction method for LTBR and STBR, primarily by reconstructing the EAC and middle ear cavities by filling them with free skin grafts. At our institution, to achieve a more favourable cosmetic outcome, the tragus is preserved when it is oncologically safe to do so. In cases where pathology specimens showed positive margins, adjuvant CCRT was administered postoperatively. Since 2017, ICT has been included as a non-operative treatment option for advanced T3 and T4 cancers (**Supplementary Figure 1**). In some instances, patients who declined standard radiation therapy were offered alternative treatments, such as CyberKnife or heavy particle therapy, and were referred to facilities capable of providing these options.

The CCRT regimen comprised 3 cycles of 80 mg/m² of cisplatin (CDDP) every 3 weeks. The ICT regimen comprised either TPF (docetaxel 60 mg/m², CDDP 60 mg/m², and 5-fluorouracil 600 mg/m²) administered in 3 cycles every 3 weeks or 80 mg/m² of paclitaxel combined with carboplatin dosed at an area under the concentration-time curve of 1.5 and cetuximab (400 mg/m² for initial dose and 250 mg/m² for subsequent doses) administered in 8 cycles. The latter regimen was introduced after 2021 to mitigate the toxicity of ICT while allowing for outpatient treatment. All patients received intensity-modulated radiation therapy, with a total planned radiation dose of either 35 fractions of 70 Gy (2 Gy per day) or 33 fractions of 66 Gy (2 Gy per day).

## 3 Results

### 3.1 Patient background and treatment details

**Figure 1:**
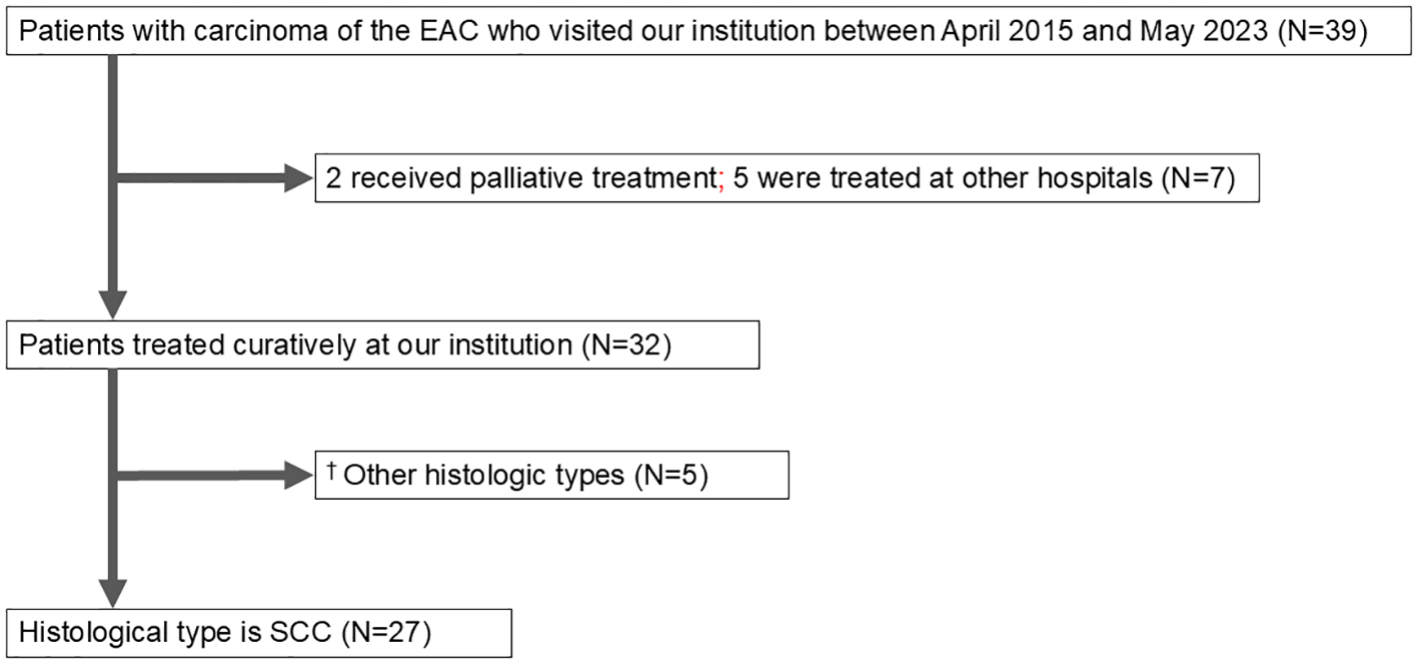
Patient flow diagram. EAC, external auditory canal; SCC, squamous cell carcinoma. ^†^The other histological types were adenoid cystic carcinoma (2), ceruminous adenocarcinoma (1), sebaceous adenocarcinoma (1), and *in situ* carcinoma (1).

Between April 2015 and May 2023, 39 patients with EAC cancer were examined at The Jikei University School of Medicine and Kashiwa Hospital. Of these, 32 patients received curative treatment in our department, 2 underwent palliative care, and 5 were treated at other institutions (3 with heavy ion therapy, 1 with CyberKnife, and 1 with radiotherapy). The histological types of carcinoma included SCC and others; specifically, there were 2 cases of adenoid cystic carcinoma, 1 case of ceruminous adenocarcinoma, and 1 case of sebaceous adenocarcinoma (**Figure 1**).

The median age of the 27 patients diagnosed with SCC of the EAC was 64 years (14 men and 13 women). The primary site of carcinoma was left in 8 cases and right in 19 cases. According to the revised Pittsburgh classification, the tumour staging was as follows: T1 in 3 cases, T2 in 4 cases, T3 in 9 cases, and T4 in 11 cases. Cervical lymph node metastasis was observed in 4 cases; 3 cases were classified as N1 and 1 case as N2b based on the Union for International Cancer Control guidelines and head and neck cancer treatment protocols. Among these patients, 13 underwent surgical treatment and 14 received non-operative management. In the surgical cohort, 1 patient underwent partial resection of the EAC, 11 patients underwent LTBR, and 1 patient underwent STBR. In the non-operative cohort, 1 patient received RT alone, 3 underwent CCRT, and 10 were treated with ICT followed by (CCRT (IC-(CCRT) (**Table 1**).

**Table 1:** Patient characteristics (N = 27).

Characteristic	No. of patients (%)
Age [years]	
Median (range)	64 (29–87)
Sex	
Male/female	14 (51.9)/13 (48.1)
Affected side	
Left/right	8 (29.6)/4 (70.4)
T category (Pittsburgh)	
1	3 (11.1)
2	4 (14.8)
3	9 (33.3)
4	11 (40.8)
N category (UICC)	
0	22 (81.5)
1	4 (14.8)
2b	1 (3.7)
Treatment	
Surgical cases	
Partial resection of the EAC	1 (3.7)
LTBR	11 (40.8)
STBR	1 (3.7)
Non-surgical cases	
(CC)RT	4 (14.8)
ICT-(CC)RT	10 (37.0)

LTBR, lateral temporal bone resection; STBR, subtotal temporal bone resection; CCRT, concurrent chemoradiation therapy; ICT, induction chemotherapy; RT, radiotherapy.

ICT followed by (CC)RT, ICT-(CC)RT.

A detailed review of the patient backgrounds was conducted for both surgical (n = 13) and non-surgical (n = 14) cases. The median age of patients who underwent surgery was 64 years (7 men and 6 women). The primary site was the left side in 3 cases and right side in 10 cases; the revised Pittsburgh classification indicated T1 in 3 cases, T2 in 3 cases, T3 in 4 cases, and T4 in 3 cases (**Table 2**). The median age of patients who did not undergo surgery was 62 years (equal sex distribution of 7 men and 7 women). The primary site was left in 5 cases and right in 9 cases. The revised Pittsburgh classification for this group indicated T1 in 0, T2 in 1 case, T3 in 5 cases, and T4 in 8 cases (**Table 2**).

**Table 2:** Treatment and outcomes (N = 27).

Patients no. (Surgical cases)	Age	Sex	T category (Pittsburgh)	N category (UICC)	Treatment	margin	Adjuvant therapy	Recurrent metastasis	Outcome
1	87	M	1	0	PR	–	–	–	NED
2	53	M	2	0	LTBR	–	–	–	NED
3	72	M	4	0	LTBR	–	–	–	NED
4	73	F	3	0	LTBR	+	RT	–	NED
5	39	F	3	0	LTBR	+	RT	Local	DOD
6	46	F	4	0	LTBR	+	–	CLN	SCB
7	40	F	1	0	LTBR	–	–	–	NED
8	57	M	2	0	LTBR	–	–	–	NED
9	29	F	2	0	LTBR	+	CCRT	–	NED
10	83	M	3	0	LTBR	–	–	–	NED
11	42	M	4	1	LTBR	–	CCRT	CLN	DOD
12	74	M	1	0	LTBR	–	–	Parotid gland	SCB
13	64	M	3	0	STBR	–	RT	–	NED
(Non-surgical cases)						ICT total course	Total CDDP (mg/m^2^)		
14	83	M	3	0	RT	–	–	Local+CLN	DOD
15	77	F	3	0	CCRT	–	240	CLN	DOD
16	65	M	4	0	CCRT	–	Unknown	Local	Unknown
17	50	F	2	0	CCRT	–	240	Local	DOD
18	44	F	4	2b	TPF(stop)→palliative RT	0	–	Local	DOD
19	57	M	4	1	ICT(TPF)-CCRT	3	240	–	NED
20	70	M	4	1	ICT(TPF)-CCRT(CBDCA)	3	CBDCA	Local	SCB
21	70	M	4	1	ICT(PCE)-RT	8	–	–	NED
22	57	M	4	0	TPF(stop)→ICT(PCE)-CCRT	8	240	Local	SCB
23	60	M	4	0	ICT(PCE)-CCRT	8	240	Local	SCB
24	67	M	4	0	ICT(PCE)-CCRT	8	240	–	NED
25	69	F	3	0	ICT(PCE)-CCRT	7	240	–	NED
26	71	F	3	0	ICT(PCE)-CCRT	8	240	Local	SCB
27	73	F	3	0	ICT(PCE)-CCRT	8	208	Local	SCB

LTBR, lateral temporal bone resection; STBR, subtotal temporal bone resection; CCRT, concurrent chemoradiation therapy; ICT, induction chemotherapy; RT; radiotherapy; CDDP, cisplatin; CBDCA, carboplatin; TPF, docetaxel + cisplatin + fluorouracil; PCE, paclitaxel + carboplatin + cetuximab; ICT-(CC)RT; ICT followed by (CC)RT; CLN, cervical lymph node; NED, no evidence of disease; DOD, died of disease; SCB, survived with cancer-bearing.

### 3.2 Treatment outcomes

The treatment outcomes are summarised in **Table 2**. Among the 13 patients who underwent surgery, nine exhibited negative margins, and four had positive margins. Of the nine patients with negative margins, seven were disease-free. Two patients with negative margins experienced recurrence: one had preoperative cervical lymph node metastasis (N1), and postoperative histopathology revealed positive extranodal involvement of the metastatic lymph node, prompting adjuvant CCRT; in the other patient, recurrence was detected in the parotid gland during postoperative follow-up, necessitating total parotidectomy as a salvage procedure, and the patient remained disease-free. All four patients with positive margins underwent LTBR. One patient received CCRT and two received RT as adjuvant therapy. One of the patients who underwent CCRT remained disease-free. One of the two patients who received RT was also disease-free; however, one patient died following disease recurrence. One patient who did not receive adjuvant therapy developed nosocomial coronavirus disease, which prevented inpatient treatment. This patient later experienced local recurrence and underwent CCRT; however, the disease progressed. Nivolumab was administered as palliative chemotherapy, resulting in a partial response, and the patient is currently a cancer-bearing survivor.

Postoperative complications were noted in one patient who underwent LTBR, mesial skull base resection, and reconstruction using a rectus abdominis skin flap. Necrosis of the flap occurred on postoperative day 14, necessitating reoperation for reconstruction with a vastus lateralis flap. The patient recovered well postoperatively and has remained disease-free.

Among the 14 patients who did not undergo surgery, the one patient who received RT alone died from the primary disease. Of the three patients who underwent CCRT and died from the primary disease, two had local recurrence and cervical lymph node involvement. Two patients treated with ICT followed by RT had unknown post-treatment outcomes. In one of these patients, the TPF regimen of ICT was discontinued because of an allergic reaction to docetaxel after the first dose; despite receiving RT, the tumour persisted, leading to death from the primary disease. Among the eight patients treated with ICT followed by CCRT, one of the two who received TPF as the ICT regimen remained disease-free, whereas the other had local recurrence, although he is currently a cancer-bearing survivor. Among the six patients treated with paclitaxel, carboplatin, and cetuximab (PCE), three experienced CTCAE grade 3 neutropenia. Two of these six patients were disease-free, whereas four had local recurrence, all of whom survived with carcinoma (**Table 2**).

**Figure 2:**
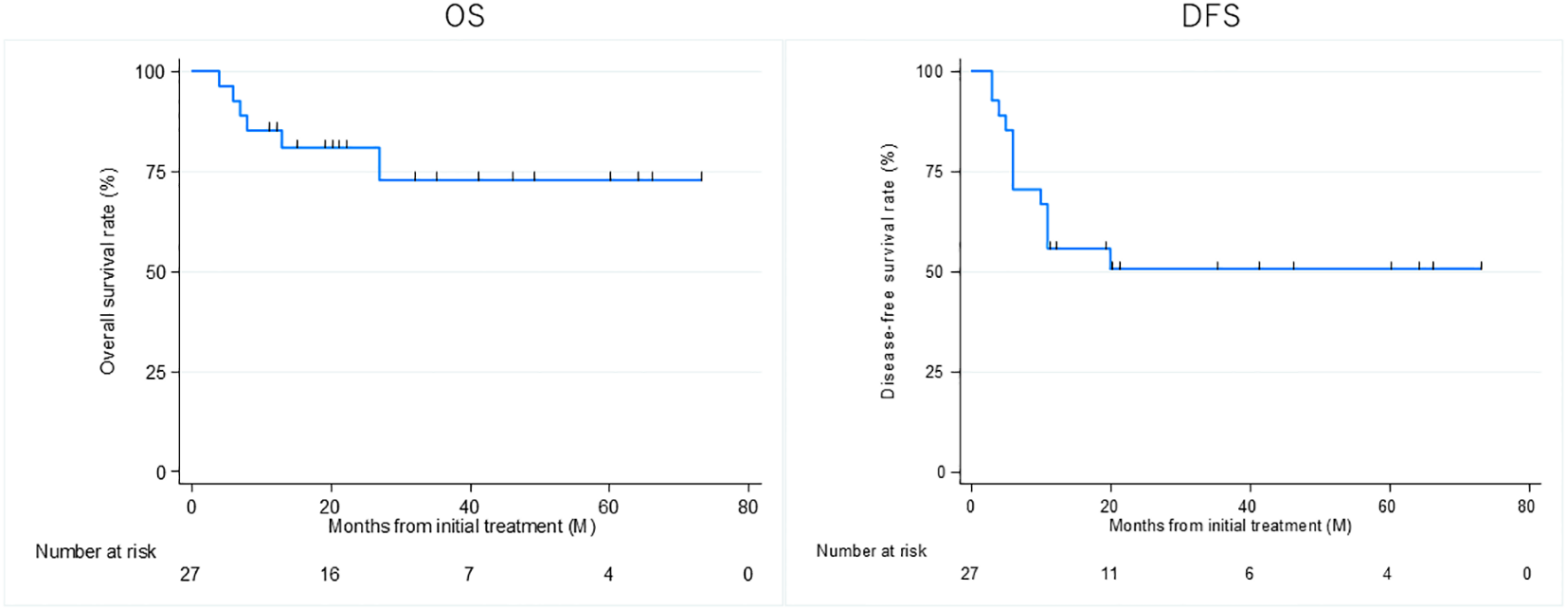
Overall survival (OS) and disease-free survival (DFS) rates of patients treated curatively for squamous cell carcinoma of the external auditory canal.

**Figure 3:**
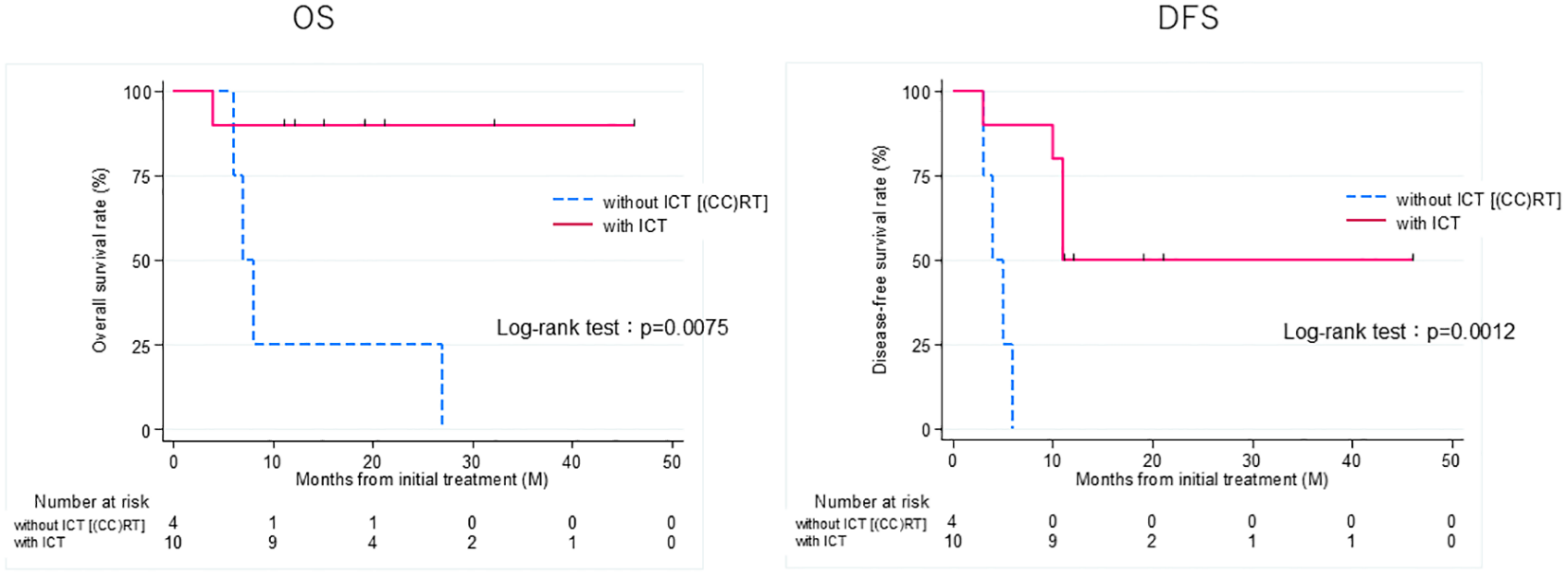
Overall survival (OS) and disease-free survival (DFS) rates of patients treated curatively for squamous cell carcinoma of the external auditory canal: with ICT vs without ICT (CCRT). CCRT, concurrent chemoradiotherapy; ICT, induction chemotherapy.

**Figure 4:**
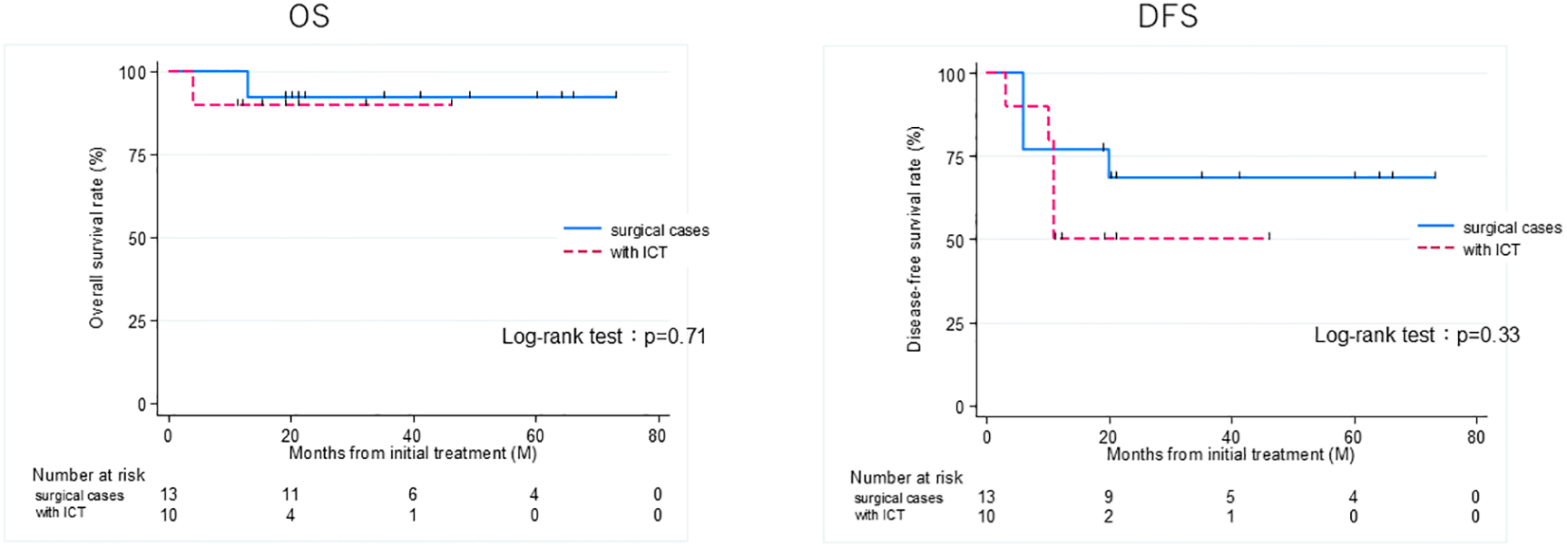
Overall survival (OS) and disease-free survival (DFS) rates of patients treated curatively for squamous cell carcinoma of the external auditory canal: surgical cases vs with ICT (non-surgical cases). ICT, induction chemotherapy.

**Figure 5:**
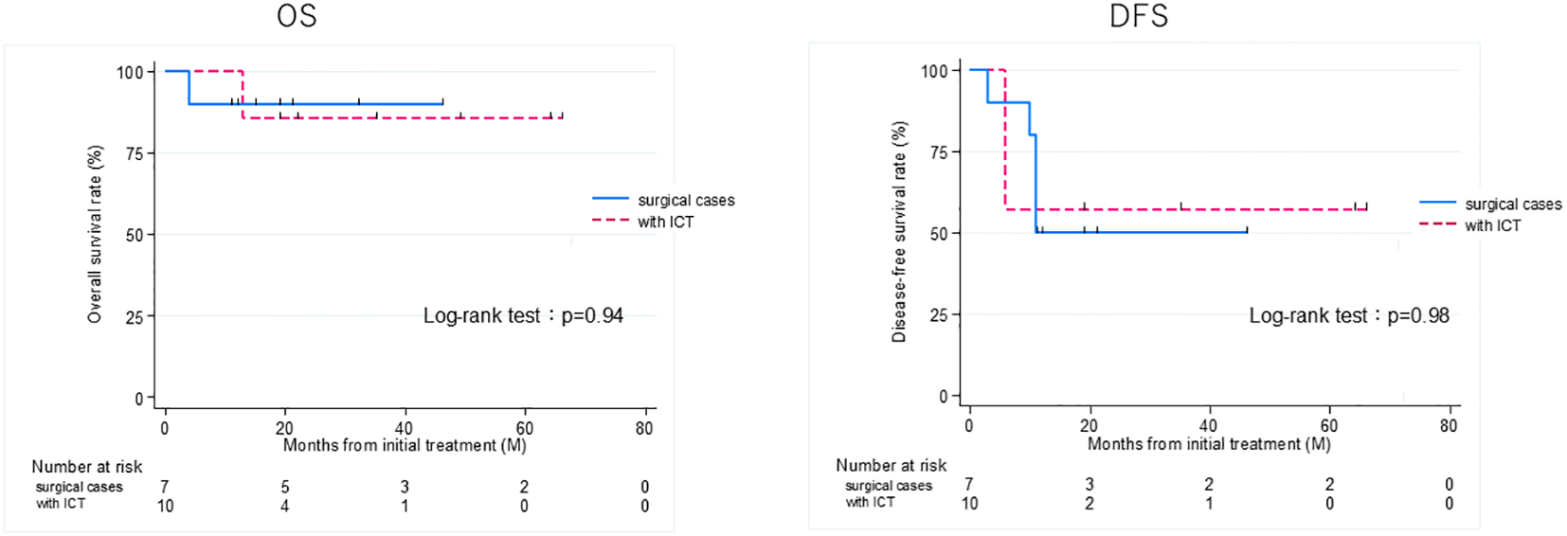
Overall survival (OS) and disease-free survival (DFS) rates of patients treated curatively for squamous cell carcinoma of the external auditory canal: surgical cases vs with ICT (non-surgical cases) in T3 and 4 group. ICT, induction chemotherapy.

Thirteen patients had recurrence, with a median time to recurrence and late metastasis of 6.0 (range, 3–20) months. Overall, the 3-year OS rate of the 27 patients with EAC SCC was 72.8% (95% CI: 46.6–87.6%), whereas the 3-year DFS rate was 50.5% (95% CI: 30.0–67.8%) (**Figure 2**). According to the revised Pittsburgh classification, the 3-year OS rates of patients in our department were 100% for T1, 50.0% for T2, 66.6% for T3, and 80.0% for T4 stages. The rates for T2 and T3 were relatively low; the T4 rate was notably high compared with those described in previous reports (**Supplementary Table 3**) (2, 12–16). Comparison of OS and DFS rates between surgical (n = 13) and non-surgical (n = 14) cases showed that surgical cases had significantly better OS rates (*p* = 0.045; HR, 6.83; 95% CI, 0.782–59.6). Although no significant difference was observed between DFS rates (*p* = 0.052, HR 2.98; 95% CI: 0.897–9.89), there was a trend toward better prognosis in the surgical cohort (**Supplementary Figure 2**). Among all patients who did not undergo surgery, for those who received CCRT, the 3-year OS and DFS rates were 90.0% and 50.0% for patients with ICT, whereas both rates were 0% for those without ICT. This result indicates a significantly better prognosis for patients who underwent ICT (OS, *p* = 0.0075; HR, 0.893; 95% CI, 0.00983–0.812; DFS, *p* = 0.0012; HR, 0.0618; 95% CI, 0.00657–0.582) (**Figure 3**). Furthermore, when comparing the groups of patients who underwent surgery and those who received ICT, there were no significant differences in either the 3-year OS or DFS rates (OS: *p* = 0.71, HR 1.678; 95% CI: 0.102–27.4; DFS: *p* = 0.33, HR 1.86; 95% CI: 0.489–7.13) (**Figure 4**). In addition, we conducted a subgroup analysis limited to patients with T3 and T4 disease to enable a more balanced comparison (**Figure 5**). No significant differences were observed in either 3-year OS or DFS rates (OS: *p* = 0.94, HR 0.903; 95% CI: 0.552–14.7; DFS: *p* = 0.98, HR 1.01; 95% CI: 0.241–4.29) (**Figure 5**). The median follow-up duration for the entire cohort was 16.7 months. When stratified by treatment modality, the median follow-up durations were 22.0 months for the surgical group, 13.6 months for the non-surgical group, 19.3 months for the ICT group, and 6.0 months for the non-ICT group.

## 4 Discussion

The degree of progression was evaluated based on the revised Pittsburgh classification and recommended surgery as the first-line treatment in operable cases, with CCRT as an adjunct therapy. In our department, the 3-year OS rate for the 27 patients with SCC of the EAC according to the revised Pittsburgh classification indicated low survival rates for patients with T2 and T3 tumours, whereas those with T4 tumours exhibited higher survival rates than the rates described in previous reports. Comparison of the OS and DFS rates between the 13 surgical and 14 non-surgical cases showed that both rates were better in the surgical group. T2, T3, and T4 cases were further categorised into surgical and non-surgical subgroups. Further, we compared our findings with those described previously to contextualise survival outcomes (12, 17).

In the surgical group, for patients with T2 tumours, LTBR was performed in 3 patients; STBR was performed in 1 of the 4 patients with T3 tumours, who have remained disease-free, whereas LTBR was performed in 3 patients. Among the 3 patients who underwent LTBR, 1 had negative margins and was disease-free, 2 had positive margins, and both received RT as postoperative treatment. One of these two patients remains disease-free, whereas the other experienced local recurrence and subsequently died from the primary disease. Among those with T4 tumours, LTBR was performed in three patients; one has remained recurrence-free. Among the two patients with positive margins, one is currently cancer-bearing, and the other with preoperative cervical metastasis (N1) experienced cervical recurrence and ultimately died from the primary disease. In patients with T3 and T4 tumours, it is essential to evaluate the feasibility of complete resection. Because of the complex anatomy of the temporal bone, the extent of tumour invasion can vary significantly, even among T3 and T4 tumour cases. Particularly, the degree of invasion into the tympanic and mastoid cavities can complicate complete surgical removal of the tumour. Several studies have indicated that patients with positive margins generally have poor prognoses (18–20). In this study, as in previous studies, the prognosis was favourable for patients complete resection, although the prognosis was poor in cases with positive margins. Only one case of T3 disease required subtotal temporal bone resection for middle ear invasion. The remaining cases were categorised as T3/T4 based on anterior or lateral extension.

In the non-surgical group, one patient with a T2 tumour was treated with CCRT; however, local recurrence occurred, and the patient ultimately died from the primary disease. Among the patients with T3 tumours, 3 were treated with ICT-CCRT, 1 with CCRT, and 1 with RT. The 3 patients treated with ICT-CCRT achieved DFS, 1 patient experienced local recurrence, and 2 patients had cancer-bearing survival. One patient with CCRT had recurrence in a cervical lymph node and died from the primary disease, and one patient with RT experienced recurrence in both regional and cervical lymph nodes and died from the primary disease, indicating a poor prognosis for patients who received CCRT and RT. Of the eight T4 cases, local recurrence was observed in one patient treated with CCRT, and the outcome remained unknown. In the remaining seven patients, treatment was initiated using ICT. One patient developed an allergic reaction, was transitioned to palliative RT, and died of the primary disease. In the other six patients, one patient underwent RT after ICT and five underwent CCRT after ICT; all patients were disease-free or were cancer-bearing survivors. Although the prognosis for patients with T4 tumours treated with CCRT is poor, ICT may be effective.

Chemotherapy for SCC of the EAC has not been established. In our institution, CDDP regimens are primarily employed during CCRT, which is in line with the practices for other head and neck cancers. However, few studies have evaluated the effectiveness of TPF regimens (21–23). Notably, CCRT with TPF for ear canal cancer is associated with fewer adverse events, such as mucositis and dermatitis (23). ICT is the treatment of choice for locally advanced head and neck cancer, offering the potential for functional preservation and improved survival. In a report of four patients with unresectable advanced carcinoma of the EAC who underwent ICT, three patients received a TPF regimen, whereas one patient received 2 cycles of ICT with a combination of paclitaxel and CDDP. According to the Response Evaluation Criteria in Solid Tumours criteria, tumours demonstrate a partial response in patients treated with TPF and stable disease in patients treated with paclitaxel and CDDP. Of those four patients, two underwent surgery, and the other two received CCRT, and two of the three survived (24). Although our results indicate a certain level of ICT efficacy, there are currently no reports demonstrating the additional benefits of ICT compared to CCRT alone, specifically for SCC of the EAC. Notably, some previous reports suggested that TPF-based chemoradiotherapy has favourable outcomes despite higher toxicity. Our findings suggest that ICT-CCRT can provide similar benefits with manageable safety profiles.

Historically, the regimen employed in ICT for head and neck cancers has primarily been TPF. However, Wang et al. (25) reported that the incidence of CTCAE Grade 3–4 adverse events associated with TPF regimens was high: 35% for neutropenia, 35% for stomatitis, 25% for anaemia, 16% for diarrhoea, and 16% for infection. Additionally, only 59% of participants completed CCRT with cisplatin as post-treatment (25). Given these adverse effects, it is important to consider factors such as age, performance status, and renal function to ensure the successful completion of treatment with TPF. In our university hospital setting, the use of TPF is not always appropriate because many patients are older and have pre-existing medical conditions. The median patient age in this study was 64 years, and only five of the 27 patients did not have pre-existing conditions. Consequently, in some instances, the highly toxic TPF regimen was unsuitable.

In non-surgical treatment of EAC carcinoma, the anatomical complexity and proximity to critical structures make TPF-based CCRT more effective than CDDP-based CCRT. Shiga et al. reported a significantly longer OS with TPF plus RT compared to CDDP plus RT for EAC SCC (21). The TPF+RT regimens used in CCRT typically involve lower doses than conventional ICT (26), which may reduce haematologic toxicity. Yamada et al. used a regimen of TPF (docetaxel 50 mg/m², CDDP 60 mg/m², and 5-fluorouracil 600 mg/m²) and reported a favourable safety profile (27), whereas another report described a regimen of TPF as appropriate (docetaxel 40 mg/m² when combined with [CDDP 70 mg/m², 5-fluorouracil 700 mg/m²]) (7). Despite these findings, reports on the use of ICT specifically for EAC SCC remain limited and are often restricted to case reports. Historically, the TPF regimen has been used for ICT in head and neck cancers, but its high toxicity frequently prevents patients from completing subsequent CRT. Moreover, the optimal reduced dose regimen of TPF for use in ICT remains unclear in the context of EAC SCC.

Therefore, the PCE regimen has received considerable attention. In a phase II study by Enokida et al. (11), the incidence of CTCAE Grade 3 adverse events associated with the PCE regimen was 11.4% for neutropenia, 8.6% for leukopenia, 5.7% for skin rash, and 5.7% for anaemia, which are lower than the rates reported for the TPF regimen. Furthermore, 97% of eligible patients completed CCRT with post-treatment CDDP. The relatively less toxic PCE regimen is associated with fewer adverse events and a higher completion rate than TPF (11). In our study, two patients experienced CTCAE grade 3 allergic reactions to TPF: one was transitioned to RT, and the other switched to a PCE regimen. Among the seven patients who underwent ICT with PCE, 42.8% experienced grade 3 neutropenia; however, all patients completed post-treatment. Four of the seven patients experienced local recurrence and continued to survive with cancer. PCE is relatively manageable even in patients with severe complications (28), suggesting that ICT with PCE is a viable option for patients who are not candidates for TPF and surgery.

In our study, the 3-year OS in the ICT group was 90%, which is favourable compared to the 5-year OS of 64.4% observed in TPF-based CCRT (14). These results suggest that ICT followed by CCRT offers similar oncologic outcomes as TPF-based CCRT. Additionally, ICT can be rapidly initiated, making it suitable for patients with rapidly progressing tumours for whom radiotherapy cannot be delayed. Furthermore, ICT may serve as a chemoselection strategy to guide the choice of definitive therapy based on treatment response.

This study has several limitations, including its single-centre design, retrospective nature, and small sample size, all of which may have influenced the accuracy and completeness of the data. The poor prognosis observed in the non-surgical group may be attributed to more advanced tumour stages compared with the surgical group. In contrast, patients treated with ICT followed by surgery exhibited outcomes comparable to those treated with surgery alone, despite the fact that the ICT group consisted exclusively of patients with T3 or T4 disease. These findings suggest a potential role for ICT in the management of locally advanced SCC of the EAC. However, further investigation is required to clarify the indications for ICT, particularly with TPF and PCE regimens. Although causal inferences cannot be made due to the study design, the results may serve as a foundation for hypotheses in future multicentre prospective trials and provide valuable observational insights that warrant longitudinal validation. We aim to continue accumulating cases to support future research.

## 5 Conclusion

This study’s results showed that at our institution, the 3-year OS and DFS rates of patients with SCC of the EAC were favourable for those who underwent surgery and those who received ICT. This study suggests that treatment outcomes can be improved by ICT in patients who do not undergo surgery.

## Data Availability

The raw data supporting the conclusions of this article will be made available by the authors, without undue reservation.
