# Supplementary material for: Treatment outcomes of squamous cell carcinoma of the external auditory canal and potential benefit of induction chemotherapy followed by chemoradiotherapy
Source: Front Oncol. 2025 Aug 15;15:1530922. doi: 10.3389/fonc.2025.1530922 (PMC12394138; doi:10.3389/fonc.2025.1530922)
Supplement: Supplementary file 1 [file DataSheet1.docx]

Supplementary Material


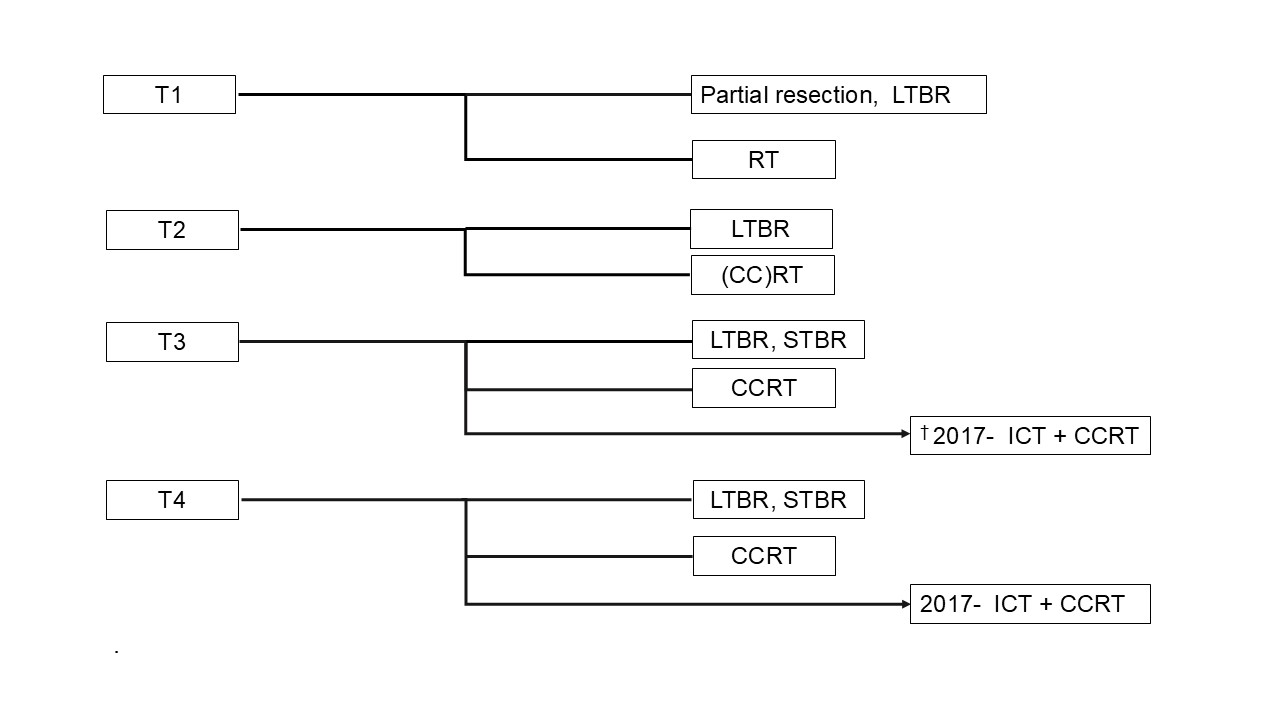


**Supplementary Figure 1.** Treatment strategies

Abbreviations: LTBR, lateral temporal bone resection; STBR, subtotal temporal bone resection;

RT, radiotherapy; CCRT, concurrent chemoradiotherapy; ICT, induction chemotherapy.

^†^ ICT has been included as a nonoperative treatment option for advanced T3 and T4 cancers since 2017

.

**
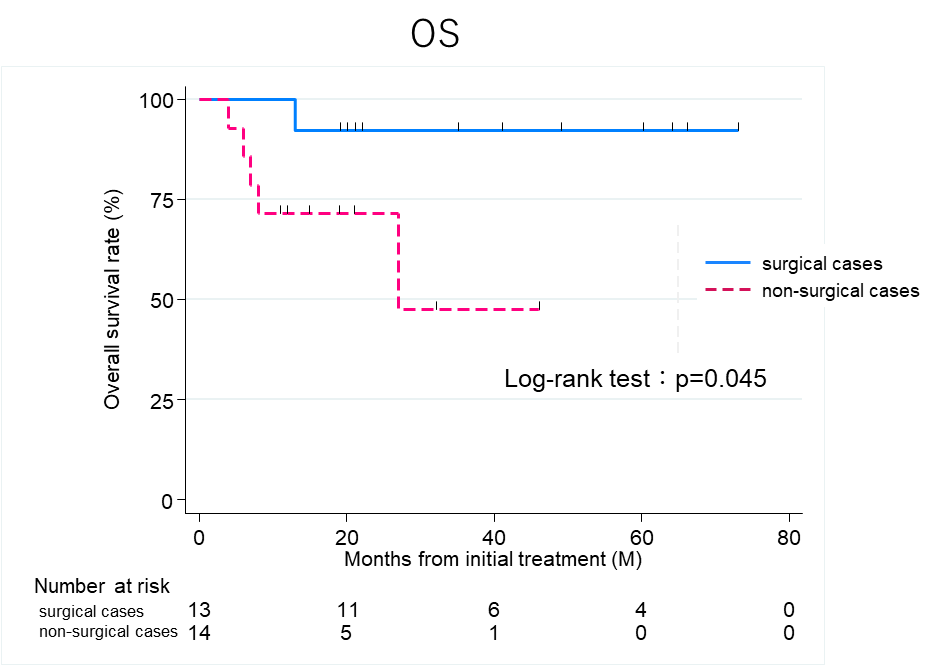

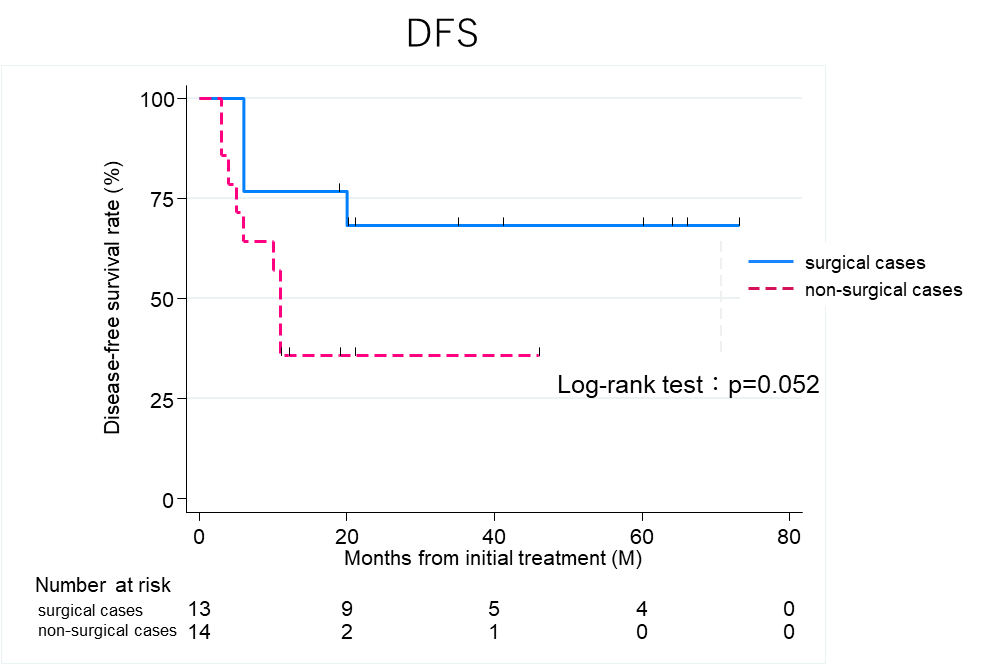
**

**Supplementary Figure 2.** Overall survival (OS) and disease-free survival (DFS) of patients treated curatively for SCC of the EAC

Surgical cases vs non-surgical cases

Abbreviations: EAC, external auditory canal; HR, hazard ratio; CI, confidence interval; SCC, squamous cell carcinoma.

**Supplementary Table 1.** Surgical patients’ characteristics (n = 13)

| **Characteristic** | **No. of patients (%)** |
| --- | --- |
| **Age** [years] | |
| Median (range) | 64 (29–87) |
| **Sex** | |
| Male/female | 7 (53.8)/6 (46.2) |
| **Affected side** | |
| Left/right | 3 (23.1)/10 (76.9) |
| **T category (Pittsburgh)** | |
| 1 | 3 (23.1) |
| 2 | 3 (23.1) |
| 3 | 4 (30.7) |
| 4 | 3 (23.1) |
| **N category (UICC)** |  |
| 0 | 12 (92.3) |
| 1 | 1 (7.7) |
| **Treatment** | |
| Partial resection of the EAC | 1 (7.7) |
| LTBR | 11 (84.6) |
| STBR | 1 (7.7) |

Abbreviations: EAC, external auditory canal; LTBR, lateral temporal bone resection; STBR, subtotal temporal bone resection

**Supplementary Table 2.** Non-surgical patients’ characteristics (n = 14)

| **Characteristic** | **No. of patients (%)** |
| --- | --- |
| **Age** [years] | |
| Median (range) | 62 (44–83) |
| **Sex** | |
| Male/female | 7 (50)/7 (50) |
| **Affected side** | |
| Left/right | 5 (35.7)/9 (64.3) |
| **T category (Pittsburgh)** | |
| 1 | 0 (0) |
| 2 | 1 (7.2) |
| 3 | 5 (35.7) |
| 4 | 8 (57.1) |
| **N category (UICC)** | |
| 0 | 10 (71.4) |
| 1 | 3 (21.4) |
| 2b | 1 (7.2) |
| **Treatment** | |
| (CC)RT | 4 (28.6) |
| ICT-(CC)RT | 10 (71.4) |

Abbreviations: CCRT, concurrent chemoradiation therapy; ICT, induction chemotherapy; RT, radiotherapy

ICT followed by (CC)RT: ICT-(CC)RT

**Supplementary Table 3.** Comparison of OS rates in this report with that in previous reports according to the revised Pittsburgh classification

| Author | Year | Number of patients |  | OS (%) | | | | |
| --- | --- | --- | --- | --- | --- | --- | --- | --- |
|  |  |  |  | All | T1 | T2 | T3 | T4 |
| This report | 2024 | 27 | 3-year OS | 72.8 | 100 | 50 | 66.6 | 80 |
| George (12) | 2021 | 437 | 5-year OS | 53.0 | 88.4 | 88.6 | 53.3 | 26.8 |
| Bingbin (13) | 2020 | 39 | 5-year OS | 78.4 | 100 | 100 | 58.9 | 50.0 |
| Ouaz (14) | 2013 | 10 | 5-year OS | - | 100 | - | 50 | 0 |
| Nakagawa (15) | 2006 | 25 | T1/2: 3-year, T3/4: 5-year | - | 100 | 100 | 80 | 35 |
| Nyrop (16) | 2002 | 10 | 2-year OS | - | 100 | - | 0 | 0 |
| Moody (2) | 2000 | 32 | 2-year OS | - | 100 | 80 | 50 | 7 |

Abbreviations: OS: overall survival
